# Strain-Promoted 1,3-Dipolar Cycloaddition of Cycloalkynes and Organic Azides

**DOI:** 10.1007/s41061-016-0016-4

**Published:** 2016-03-22

**Authors:** Jan Dommerholt, Floris P. J. T. Rutjes, Floris L. van Delft

**Affiliations:** 1 Radboud University, Nijmegen, The Netherlands; 2 Wageningen University and Research Centre, Wageningen, The Netherlands

**Keywords:** Strain-promoted cycloaddition, Cyclooctyne, BCN, DIBAC, Azide

## Abstract

A nearly forgotten reaction discovered more than 60 years ago—the cycloaddition of a cyclic alkyne and an organic azide, leading to an aromatic triazole—enjoys a remarkable popularity. Originally discovered out of pure chemical curiosity, and dusted off early this century as an efficient and clean bioconjugation tool, the usefulness of cyclooctyne–azide cycloaddition is now adopted in a wide range of fields of chemical science and beyond. Its ease of operation, broad solvent compatibility, 100 % atom efficiency, and the high stability of the resulting triazole product, just to name a few aspects, have catapulted this so-called strain-promoted azide–alkyne cycloaddition (SPAAC) right into the top-shelf of the toolbox of chemical biologists, material scientists, biotechnologists, medicinal chemists, and more. In this chapter, a brief historic overview of cycloalkynes is provided first, along with the main synthetic strategies to prepare cycloalkynes and their chemical reactivities. Core aspects of the strain-promoted reaction of cycloalkynes with azides are covered, as well as tools to achieve further reaction acceleration by means of modulation of cycloalkyne structure, nature of azide, and choice of solvent.

## Introduction

The spontaneous reaction of cycloalkynes with an organic azide, in all its simplicity, is a fascinating organic chemical transformation. Simply by mixing and stirring, without the necessity of reagents, catalysts, or carefully controlled reaction conditions, a stable triazole product is formed by fast and selective cycloaddition of cycloalkyne with azide. As will become clear throughout this chapter, the latter reaction has now firmly established itself as a powerful and versatile chemical process with broad academic and commercial applications. Core to the chemistry lies a highly strained, medium-sized cyclic alkyne, most prominently a cyclooctyne. In this chapter, the synthesis and chemical reactivity of cyclic alkynes is broadly delineated, with particular emphasis on undoubtedly the most important of applications of cycloalkynes: cycloaddition with an organic azide, leading to the formation of a stable triazole.

## The Fascinating Chemistry of Cycloalkynes

### Conception of Cycloalkynes

In the second half of the previous century, interest emerged at several laboratories around the world to explore the synthesis and properties of medium-sized cycloalkynes. Pioneers in the field, Blomquist et al., at Cornell University (USA), convincingly demonstrated in 1951 that plain cyclononyne and cyclodecyne could be accessed by oxidative decomposition of the respective cycloalka-1,2-diones, and isolated in pure form by distillation [7]. Two years later, the same group also reported the successful preparation of the eight-membered ring acetylene [8], while similar explorations on cycloalkynes were reported by Prelog and colleagues at the ETH in Zurich (Switzerland) around the same time [44]. It must be noted that the preparation and isolation of cyclooctyne had been claimed by Domnin (USSR) some 15 years earlier [21], but the reported characterization data suggest that the compound was—at best—obtained as a mixture with the isomeric 1,2-cyclooctadiene and other unsaturated hydrocarbons. In 1961, it was Wittig at the University of Heidelberg (Germany) who was the first to demonstrate the formation of the yet-smaller five-, six-, and seven-membered cycloalkynes, as well as 1,2-dehydrobenzene, better known as benzyne [62].

The successful preparation of cycloalkynes also opened up the possibility to explore their unique chemical reactivity. In fact, the transient existence of the cycloalkyne species could initially only be indirectly corroborated by fast in situ trapping of the smaller-sized rings (seven carbons and below) before decomposition [31]. While not strictly applicable to cyclooctyne, which is the smallest cyclic alkyne that can be isolated and stored in pure form, Blomquist already noted that nevertheless careful exclusion of air was requisite to avoid rapid decomposition. More importantly, he was also the first to observe that “cyclooctyne reacts explosively when treated with phenyl azide, forming a viscous liquid product” [8]. This remark is in fact the first historic administration of a process that has now become known as strain-promoted azide–alkyne cycloaddition (SPAAC).

### Synthetic Preparation of Cycloalkynes

A range of different synthetic procedures is known to obtain medium-sized cycloalkynes, as covered by several reviews [38]; Tochtermann [25]. In this paragraph, only the most relevant procedures will be covered.

**Fig. 1:**
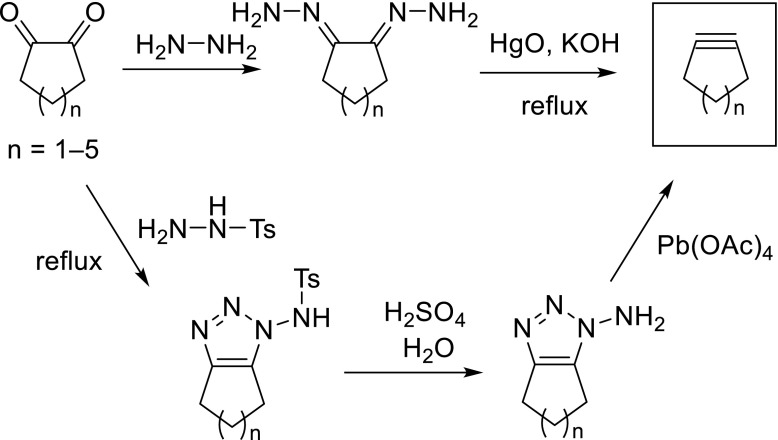
Oxidative decomposition of cycloalka-1,2-dione dihydrazone, leading to medium-sized cycloalkynes (*n* = 1–5)

The first synthetic reports on cycloalkynes involved a base-catalyzed oxidative decomposition of bis-hydrazones, readily prepared from the respective precursor 1,2-cycloalkadiones by condensation with hydrazine (Fig. 1, top). As oxidant, mercury oxide is most typically applied, however Ag_2_O or Pb(OAc)_4_ are also suitable. An analogous procedure (Fig. 1, bottom) employs the tosylate (Ts) derivative of hydrazine, which upon condensation with the diketone under reflux conditions forms the tosylated aminotriazole intermediate. Acidic removal of the tosyl group, followed by lead-mediated oxidation, also forms the desired cycloalkyne ring.

A direct procedure to obtain a cycloalkyne from a cycloalkanone involves the conversion into semicarbazone, followed by oxidation with selenium dioxide. The resulting intermediate 1,2,3-selenadiazole can be isolated in pure form and will eliminate, upon heating to 170–220 °C (or refluxing ethylene glycol), elemental selenium and nitrogen, with formation of the cycloalkyne.

**Fig. 2:**
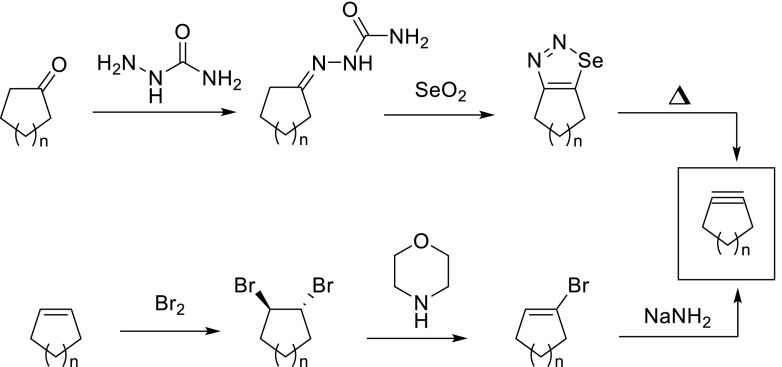
Formation of cycloalkynes by fragmentation of 1,2,3-selenadiazole (*top*) or dehydrohalogenation (*bottom*)

The most often applied and most reliable procedure to obtain a cycloalkyne (Fig. 2, bottom), as originally developed by Brandsma in the Netherlands, [11], involves the stepwise double dehydrohalogenation of a vicinal dihalogenide (typically bromide), which can be readily obtained from a cyclic alkene upon treatment with elemental halogen. In this case, a first E2-elimination may take place under mildly basic conditions, forming an intermediate alkene, which in turn will undergo a second elimination upon treatment with a strong base like sodium amide or potassium *tert*-butoxide. More conveniently even, in many cases both eliminations can be induced in a one-step process upon treatment with a large excess of a strong base and/or heating.

Some more exotic synthetic processes for the synthesis of cycloalkynes, including reductive dehalogenation, photolytic elimination of bis-hydrazones or fragmentation of α,β-epoxyketones have also been reported, but are beyond the scope of this chapter.

### Reactions of Cycloalkynes

**Fig. 3:**
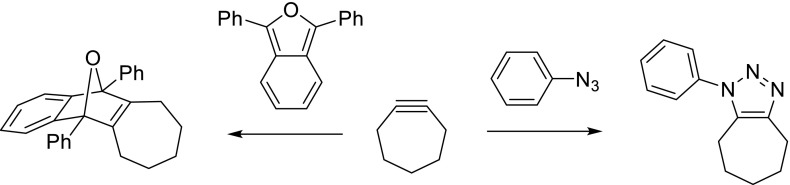
Reaction of cycloheptyne with 1,3-diphenylisobenzofuran or phenyl azide, leading to bicyclic oxanorbornene (*left*) or phenyltriazole (*right*), respectively

The stability of cyclic alkynes rapidly decreases with ring size. In fact, stability is directly correlated with the C–C C–C bond angle, which, by virtue of the cyclic structure, cannot adopt the ideal 180° bond angle of sp-hybridized carbon atoms. Interestingly, cyclooctyne was identified as the smallest isolable cycloalkyne, although its acetylene bond angle of 163° still significantly deviates from linear. The experimentally determined ring strain of cyclooctyne is ~18 kcal/mol [58], compared to 12.1 kcal/mol for saturated cyclooctane [6]. Not surprisingly, ring-strain is accountable for the intrinsically low stability of medium-sized cyclic alkynes, with calculated ring strains of 25 kcal/mol and above [6], leading to fast degradation and/or polymerization of seven-membered and smaller cycloalkynes, and thwarting their isolation in pure form. Gratifyingly, the same ring strain also imparts a unique reactivity profile onto medium-sized cycloalkynes, which may be advantageously employed in many ways as described here. In fact, the first synthetic preparation of seven-, six-, and five-membered cycloalkynes by Wittig et al. in [62] could only be corroborated by in situ generation of the formed alkyne in the presence of a suitable ‘alkynophile’ for fast (4 + 2) cycloaddition [62]. In particular, 1,3-diphenylisobenzofuran (as illustrated for cycloheptyne in Fig. 3, left) was used, forming a stable oxanorbornene (4 + 2)-cycloadduct, suitable for isolation and characterization. An alternative quenching reagent for small-ring cycloalkynes is phenyl azide (Fig. 3, right), which will form a stable aromatic triazole by (3 + 2)-dipolar cycloaddition.

**Fig. 4:**
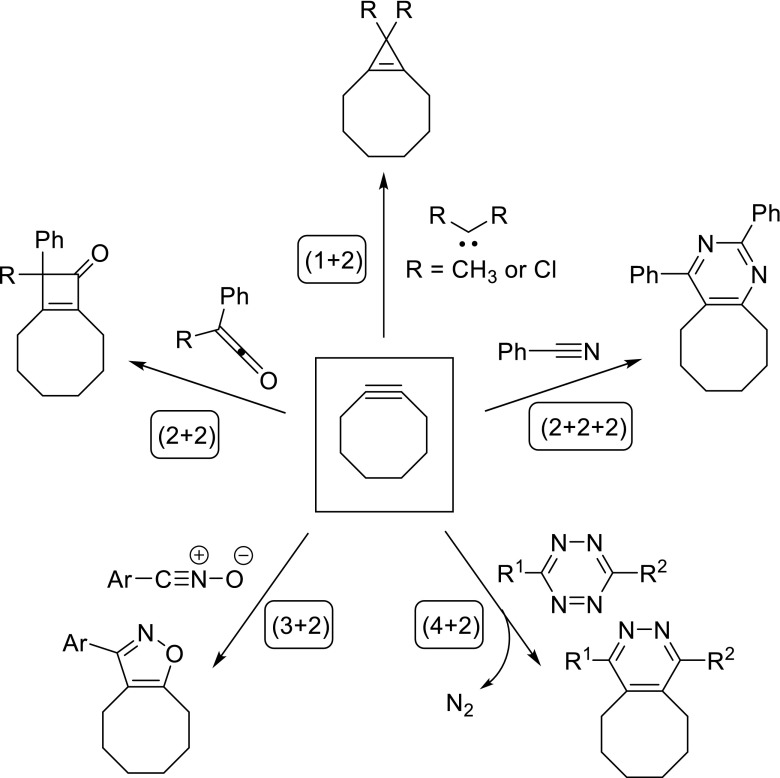
Select examples of cycloaddition reactions of cyclooctyne

Since these first reports on the reactions of cycloalkynes with isobenzofuran and phenyl azide, a wide range of other alkynophile cycloaddition partners have become known, as conceptually illustrated with model compounds in reactions with cyclooctyne in Fig. 4. It becomes immediately clear that cycloalkynes can undergo a wide range of cycloaddition reactions, including (1 + 2), (2 + 2), (2 + 2 + 2), (3 + 2), and (4 + 2) cycloadditions. Besides these, more exotic transformations like (6 + 2) cycloadditions, hydrogen transfer reactions, radical additions, and reactions with metal salts or complexes, are also known. For further information on this topic, the reader is referred to several earlier reviews [25, 32].

### Dipolar Cycloaddition of Cycloalkynes with Azides

The prototypical example of the reactivity of cycloalkynes in organic chemistry transformations, as already noted in the first report on the isolation of pure cyclooctyne [8], is the (3 + 2) dipolar cycloaddition with organic azides. A logical explanation for the fast and spontaneous reaction of cycloalkynes with (phenyl)azide therefore lies in the highly favorable enthalpic release of ring-strain, by going from a strained ring to a fused ring system with favorable bond angles for the sp^2^-hybridized carbon atoms of the resulting triazole. It has been calculated [6] that the barrier of activation for (3 + 2) cycloaddition is directly correlated to strain energy of cycloalkynes. Houk et al. performed calculations on the transition state of the Huisgen 1,3-dipolar cycloadditions of phenyl azide with acetylene and cyclooctyne with density functional theory at the B3LYP level [22]. The low activation energy of the cyclooctyne cycloaddition (Δ*E* ^‡^ = 8.0 kcal/mol) compared to the strain-free acetylene cycloaddition (Δ*E* ^‡^ = 16.2 kcal/mol) was explained due to the decreased distortion energy for cyclooctyne to reach the requisite C–C–C bond angle of 158°–166° in the transition state versus the linear alkyne, i.e., deformation from 153° and 180°, respectively. Given the alkyne ring strain, the reaction of an organic azide with a cyclic alkyne, typically cyclooctyne, has become commonly known as strain-promoted azide–alkyne cycloaddition (SPAAC).

## Speeding Up SPAAC

### Copper-Free Click Reaction

With most of the activity around addition reactions of cyclic alkynes taking place in the last century, interest in this particular subclass of chemistry presumably would have slowly subsided if not for the clever insight by researchers at the University of California, Berkeley [1] that strain-promoted cycloaddition of cyclooctynes with azides is a highly versatile copper-free version of the popular click reaction [46, 57]. It was reasoned that remote attachment of a suitable functional handle to the cyclooctyne would enable the smooth conjugation to any organic azide, in any solvent of choice. In particular, it had become clear that the use of the copper-catalyzed azide–alkyne cycloaddition (CuAAC) was severely compromised in the context of biological matter, due to the toxicity of the inevitable copper(I)-species to living cells and organisms. In a seminal paper [1], it was shown that chemical functionalization of cyclooctyne with (+)-biotin enabled clean and selective visualization of living cells with azidosugars metabolically incorporated on the cell surface glycans, upon subjecting the cells to a SPAAC protocol with biotin-cyclooctyne followed by fluorescent labeling with a streptavidin-fluorophore. The broad impact of this seminal application of SPAAC, referred to in popular terms as “copper-free click reaction”, can hardly be overestimated.

### The Quest for More Reactive Cycloalkynes

**Fig. 5:**
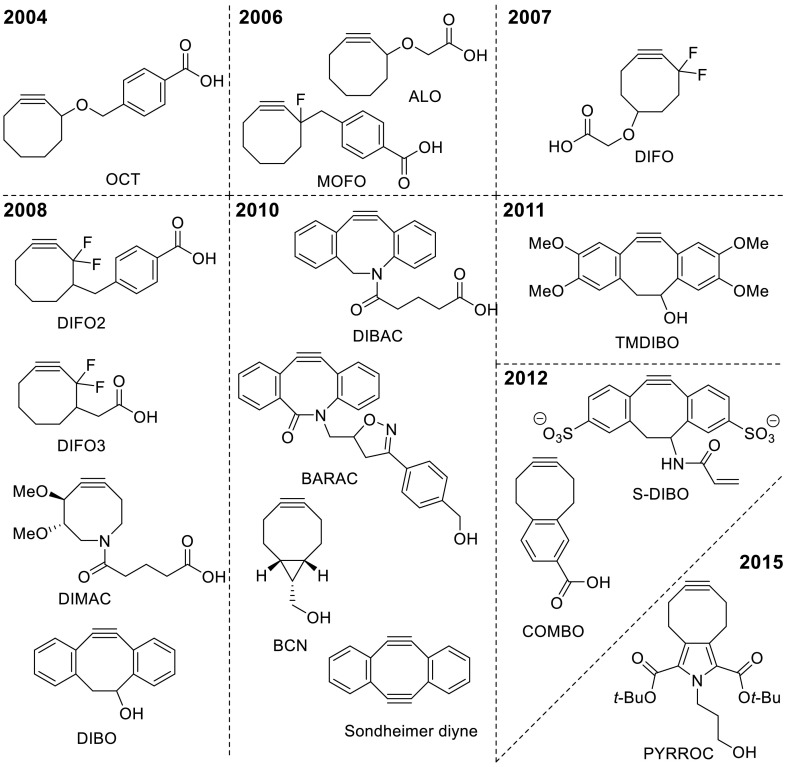
Overview of functionalized cyclooctynes suitable for conjugation reactions, grouped by year of discovery. *OCT* cyclooctyne, *MOFO* monofluorinated cyclooctyne, *DIFO* difluorocyclooctyne, *DIMAC* dimethoxyazacyclooctyne, *DIBO* dibenzocyclooctyne, *DIBAC* dibenzoazacyclooctyne, *BARAC* biarylazacyclooctynone, *BCN* bicyclononyne, *TMDIBO* 2,3,6,7-tetramethoxy-DIBO, *S-DIBO* sulfonylated DIBO, *COMBO* carboxymethylmonobenzocyclooctyne, *PYRROC* pyrrolocyclooctyne

While the first application of SPAAC rapidly found its way also outside the field of metabolic labeling, it also became quickly apparent that the relatively slow reaction kinetics required large excesses of reagents, long incubation times, and led to relatively little signal. In fact, visualization of metabolic labeling of azido-modified living cells was initially even less efficient than by Staudinger–Bertozzi ligation [49], a process known to suffer from poor reaction kinetics as well as oxygen sensitivity of the phosphine probe. As a result, a range of laboratories around the world, including ours, have embarked on the synthesis and evaluation of new cycloalkyne probes with the aim of lifting the reactivity without compromising on stability. A comprehensive overview of functionalized cyclooctynes that have been developed in the past decade, categorized by year of discovery, is provided in Fig. 5.

In general, two classes of cyclooctynes can be recognized: the earliest generation aliphatic cyclooctynes, and (di)benzoannulated cyclooctynes. The first dibenzoannulated cyclooctyne (DIBO) suitable for conjugation, developed by Ning et al. [40], has led the field to more reactive probes. It is commonly understood that the enhanced reactivity of (di)benzoannulated cyclooctynes is caused by the increase in ring strain imparted by the multiple sp^2^-hybridized carbons. The latter phenomenon can also account for the reactivity order BARAC > DIBAC > DIBO, given that the number of sp^2^-hybridized atoms in the ring decreases from 6 to 4, respectively. BARAC is in fact an interesting example of the fine balancing act between reactivity and stability that comes along with the development of cyclooctyne probes: BARAC displays a reaction rate constant of nearly 1 mol^−1^ s^−1^ (see Table 1), but unfortunately is inherently unstable and rapidly decomposes [28]. Two cyclooctyne probes difluorobenzocycloocytne (DIFBO [55]) and 3,3,6,6-tetramethylthiaheptyne (TMTH [5]) are yet more reactive than BARAC but cannot be isolated in pure form before rapid decomposition takes place (not depicted in Fig. 5). Efforts from our own laboratory led to the development of DIBAC [16], a cyclooctyne that combined excellent stability with a reaction rate constant that is top among the family members. Interestingly, Kuzmin et al. [33] and Campbell-Verduijn et al. [13] rapidly followed with publications of the same molecule, albeit obtained by different synthetic paths and with different given names (ADIBO and aza-DBCO, respectively). In this chapter, the original term DIBAC will be consistently used to denote this azacyclooctyne structure, although DBCO is also often applied.

**Table 1:** Reaction rate constants (for BnN_3_ or similar aliphatic azide) and synthetic accessibility of practically stable cyclooctynes

Entry	Cyclooctyne	*k* (×10^−3^ M^−1^ s^−1^)	Solvent	#Steps	Yield (%)
1	OCT	2.4	A	4	52
2	DIMAC	3.0	A	11	5
3	MOFO	4.3	A	5	15
4	PYRROC	6.0	A	11	3
5	Sondheimer	8.8	C	4	41
6	DIFO2	42	A	8	27
7	DIFO3	52	A	10	21
8	DIFO	76	A	10	1
9	TMDIBO	94	C	7	57
10	S-DIBO	112	C	7	13
11	DIBO	120	B	5	10
12	BCN	140	C	4	15
13	COMBO	235	A	6	11
14	DIBAC	310	C	9	41
15	BARAC	960	A	6	18

Cyclooctynes are listed in order of reactivity. Solvent: A = CD_3_CN, B = CD_3_CN:D_2_O (3:1), C = CD_3_OD or CH_3_OH

Besides reactivity, which is typically determined in organic (co)solvents like MeOH or MeCN, two other factors that qualify a given cyclooctyne, namely lipophilicity and size, are of high importance. For example, benzoannulation has shown to be beneficial for cyclooctyne reactivity but inevitably also leads to concomitant enhanced steric interactions and lipophilicity, which is typically not beneficial when SPAAC in water is envisaged. The first attempt to address the issue of lipophilicity was made by introduction of methoxy-groups on the cyclooctyne ring, as in DIMAC [54]. Although displaying excellent water solubility, reactivity of DIMAC was also severely compromised. Several more hydrophilic variants of DIBO have also been developed over the years, most prominently TMDIBO [56] and S-DIBO [23], both of which show much improved solubility in water, but the reaction rate constants remain rather low, as for the parent DIBO structure. The cyclooctynes COMBO [60] and PYRROC [24] have most recently been developed and are characterized by an intermediate, monobenzoannulated structure. Not unexpectedly, the reaction rate constants of COMBO and PYRROC are also lower than those for the analogous dibenzoannulated structures (Table 1). One notable exception to the rule that benzoannulation is a necessity to achieve acceptable reactivity is BCN, developed in our own laboratory [19]. For BCN, reactivity is induced by ring fusion of cyclooctyne to cyclopropane, leading to the typical bicyclo[6.1.0]non-4-yne structure. Although less reactive than DIBAC, reaction rate constants for the *endo*-isomer are still among the highest in the pack (see Table 1), while the synthesis of BCN is exceptionally short and simple.

From Fig. 5, it also becomes apparent that while active development of cyclooctynes took place in the years 2008–2010, the intensity in the field has more or less subsided in the past years. One possible reason for this observation may be found in the fact that further boosting of the reactivity of cyclooctyne for azide is typically penalized by loss in stability, as earlier mentioned for BARAC, DIFBO, and TMTH. Another explanation lies in the current commercial availability of the cyclooctynes DIBO, DIBAC, and BCN, the three of which have dominated the field of SPAAC in the past years.

### Influence of Azide Structure on Reaction Rate

**Fig. 6:**
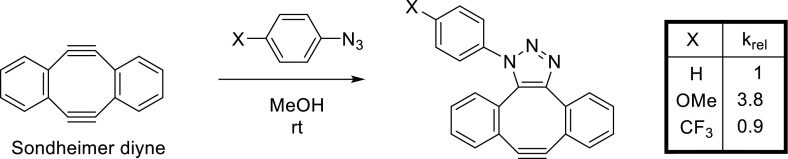
Negligible influence of azidobenzene *para*-substitution on reaction rate with dibenzoannulated cyclooctyne

While significant effort has been devoted over the years to the development of more reactive cyclooctynes, as delineated above, only scant investigations so far have focused on the increase of SPAAC rates by modulation of the complementary component, i.e., the azide. In fact, the vast majority of reported applications of SPAAC are based on reaction with simple aliphatic azides. As a logical consequence, reaction rate constants are also nearly always determined with an aliphatic azide (typically benzyl azide), but seldom with an aryl azide. One possible reason that aromatic azides are generally avoided for SPAAC may lie in a report that *p*-azidophenylalanine shows sevenfold lower reactivity than that of the corresponding aliphatic azidomethyl analogue [66], at least in conjunction with DIBAC, a dibenzoannulated cyclooctyne. Furthermore, the observation that reaction rates of aromatic azides are hardly influenced by changing the electronic nature of substituents (as determined for *p*-methoxy and *p*-CF_3_-phenyl azide, see Fig. 6) may have provided further ground to avoid aromatic azides for SPAAC [64]. These observations obviously support the notion that aryl azides, and in particular electron-poor azides (as in *p*-CF_3_-PhN_3_), are better avoided in case high SPAAC reaction rates are desirable. Other studies have reported a reactivity enhancement (up to 2.2× faster) upon the introduction of electron-withdrawing halogen substituents on DIFO [4], BARAC [28] and DIBAC [14, 18], all of which suggests that the SPAAC mechanism primarily proceeds via HOMO_azide_–LUMO_cyclooctyne_ interaction so that electron-rich azides are preferred. Interestingly, it has been known since the 1960s that electronegative substituents on aryl azides accelerate reaction rates with strained alkenes, for example a fourfold reaction rate enhancement of *p*-nitro substitution of phenyl azide with norbornene [50]. Even more markedly, picryl azide was found to react with norbornene almost 1000 times faster than phenyl azide [2].

**Fig. 7:**
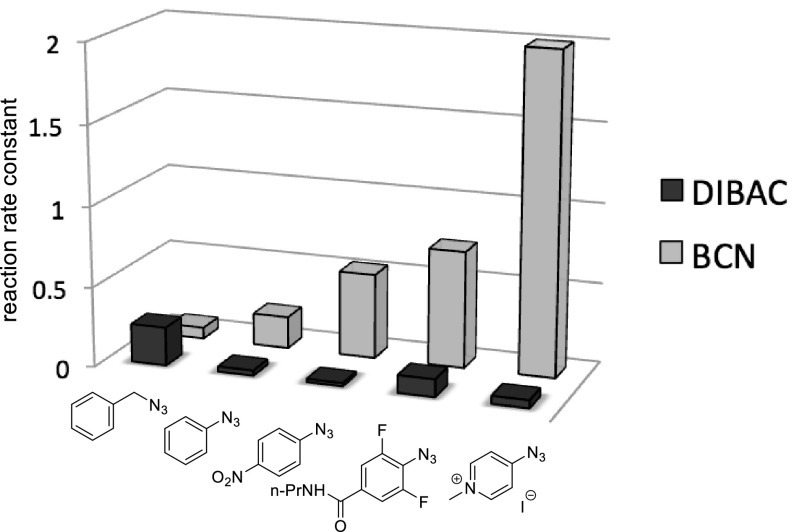
Relationship between nature of the azide, structure of cyclooctyne and reaction rate constants. Reaction rate constants were determined in CD_3_CN:D_2_O = 3:1

Based on these observations, we recently concluded that the apparent slower reaction of electron-poor azides in SPAAC only holds for benzoannulated cyclooctynes (with low-lying LUMO), while in combination with more electron-rich cyclooctynes (like BCN), azides can react by a second, inverse electron-demand mechanism (i.e., SPAAC) as well [20]. As a result, it was first found that strain-promoted cycloaddition of aromatic azides with BCN is nearly eight times faster than with DIBAC, which is the opposite trend for reaction with benzyl azide. Introduction of electron-withdrawing substituents on the aryl azide led to a further acceleration in reaction with BCN, while reaction rate with DIBAC stayed more or less constant. The highest reaction rate acceleration was achieved with a pyridinium derivative, giving an almost 30-fold by faster reaction than benzyl azide and an absolute rate constant of almost 2 M^−1^ s^−1^ (Fig. 7).

### Solvent Effects

Since Breslow first pointed out the effect of water on cycloaddition reactions [45], an extensive amount of kinetic measurements in the field have been determined, in particular focusing on the Diels–Alder reaction, a (4 + 2)-cycloaddition reaction. Studies of the comparative rates of 1,3-dipolar cycloaddition reactions in water and organic solvents have not attracted the same level of attention. Some work in the field has focused on reactions of transient nitrile oxides [59] and nitrilimines [39] generated in aqueous environments and there are some reports on rate measurements on 1,3-dipolar cycloaddition reactions in water–organic mixtures. Recently, it was reported that with an increasing mole fraction of water, significant enhancement of 1,3-dipolar cycloaddition rates occurs [12]. It was suggested that a dominant hydrogen-bonding effect operates in water-induced rate enhancements of 1,3-dipolar cycloaddition reactions. The hydrogen-bonding effect involves secondary hydrogen bond bridging from the primary water-solvation shell of the transition state and the growth of structured water clusters, which was also supported by theoretical calculations.

The awareness that increasing levels of water translate into faster reaction rates has received surprisingly little attention in the field of SPAAC, despite the fact that a large number of applications involve conjugation processes on natural biomolecules (peptides, proteins, glycans, oligonucleotides) in aqueous systems. Nevertheless, classification and appreciation of a given cyclooctyne probe is typically only performed by determination of reaction rate constants in any of a range of solvents like methanol, acetonitrile or water (or deuterated versions thereof if performed in NMR, vide infra), or mixtures thereof. For example, Table 1 above displays the reaction rate constants of the known cyclooctynes across these solvents, which in fact makes it difficult to truly compare the usefulness of these probes in a quantifiable manner. Furthermore, it has been noted by several authors that SPAAC proceeds (significantly) faster in more aqueous solvent systems [9, 19, 24, 40, 60].

For these reasons, we embarked on a study to determine the reaction rate constants of the most commonly applied dibenzoannulated and aliphatic cyclooctynes, respectively DIBAC and BCN, as deduced from a SciFinder^®^ structure search of these compounds. As becomes clear, since the year of its inception (2010), DIBAC has made its appearance in 260 unique publications (184 scientific publications and 80 patent applications) while BCN is reported 155 times (103 papers and 51 patent application). For comparison, DIBO, the third-most popular cyclooctyne gave 135 unique hits for the same time-frame (74 papers, 42 patent applications). Moreover, given the lower reaction rate constant of DIBO versus DIBAC, as well as their large structural similarity, DIBO was omitted from the studies below.

It was decided to evaluate both DIBAC and BCN with a range of representative azides and in three different solvent systems: MeOD, CD_3_CN:D_2_O (1:3), both for NMR measurements, and THF:H_2_O (9:1), for quantification by IR. In order to ensure that both probes and azides would be soluble in the acetonitrile–water mixture, a hydroxyethylated derivative was prepared in several instances. The same strategy was applied to some of the more lipophilic aromatic azides, which led to full solubility except for the diisopropylated azidobenzene derivative (entry 5), where NMR was performed in a MeCN:H_2_O (1:2) mixture.

Several interesting observations can be made from Table 2. First of all, the picture is confirmed that DIBAC is faster than BCN in reaction with aliphatic azides (entries 1–3). While apparent in all cases, the reaction rate difference is markedly larger in MeOD or CD_3_CN:D_2_O (factor ~10) than in THF:H_2_O (factor ~3). Also striking is the rate constant of 1.9 M^−1^ s^−1^ for reaction of DIBAC with benzyl azide (entry 1), which is more than double than that for the other aliphatic azides (entries 2 + 3). We attribute this to the relatively low solubility of benzyl azide in an aqueous solvent system, resulting in faster reaction by means of a hydrophobic effect. The hydrophobic effect may also be accountable for another interesting observation, that the reactions of DIBAC with aliphatic azides are much faster in 75 % aqueous acetonitrile than in methanol or in 10 % aqueous THF.

**Table 2:** Reaction rate constants for cycloadditions of aliphatic (entries 1–3) and aromatic (entries 4–6) azides with BCN and DIBAC in different solvent systems

Entry	Azide structure						
		MeOD	D_2_O:CD_3_CN (3:1)	H_2_O:THF (1:9)	MeOD	D_2_O:CD_3_CN (3:1)	H_2_O:THF (1:9)
1		0.03	0.20	0.12	0.42	1.9	0.36
2		0.04	0.07	0.05^a^	0.33	0.90	0.21^a^
3		0.03	0.09	0.05^a^	0.29	0.82	0.20^a^
4		0.18	0.75	0.30	0.11	0.11	0.08
5		0.65	1.5^b^	0.30^a^	2.3	~4^b,c^	0.77^a^
6		1.1	~6^c^	0.73^a^	0.18	0.95	0.11^a^

Rate constants in MeOD and D_2_O:CD_3_CN were determined by NMR, in H_2_O:THF by IR

^a^Data taken from similar compounds, as reported earlier [20]

^b^In D_2_O:CD_3_CN (2:1)

^c^Estimated based on known ratio BCN:DIBAC [20]

As expected, the reactivity trend is reversed for azidobenzene (entry 4), where BCN is up to seven times faster than DIBAC (in 75 % aqueous CD_3_CN), with a reaction a rate constant of 0.75 M^−1^ s^−1^. As reported earlier [64], introduction of an *ortho*-isopropyl group on azidobenzene (entry 5) leads to significant rate acceleration for reaction with DIBAC, which is already very high in MeOD (2.3 M^−1^ s^−1^) and too fast to measure in D_2_O:CD_3_CN by NMR. The number provided in the Table (~4 M^−1^ s^−1^) for this solvent system is instead derived by multiplying the rate constant determined for BCN (1.5 M^−1^ s^−1^) by the earlier determined relative reaction rate DIBAC:BCN = 2.5 [20]. A similar strategy estimates the reaction rate constant of BCN with the electron-poor difluorinated phenylazide (~6 M^−1^ s^−1^, entry 6) from the value experimentally determined for DIBAC.

### Tools to Quantify SPAAC Reaction Rates

The main determinant of the quality of any given cyclooctyne for SPAAC reaction is its reaction rate constant with azide (aliphatic or aromatic). Throughout the years, a large number of reaction rate constants have been experimentally determined for different cycloalkynes and azides, mainly by four analytical techniques: (1) NMR, (2) UV spectroscopy, (3) IR spectroscopy, and (4) fluorescence.

The most commonly applied method to determine a SPAAC reaction rate constant is by NMR [1]. To this end, a cyclooctyne and an azide are mixed in a deuterated solvent and formation of product is quantified by integration of diagnostic peaks of the formed triazole product. Given the fact that the triazole ring itself is fully substituted, other diagnostic protons in the product with a unique, non-overlapping shift in the NMR spectrum, must be identified (or specifically introduced in one of the substrates). Alternatively, one or more distinct proton peaks of starting material can be integrated. It must be noted that the formation of a mixture of regioisomeric triazoles, as is typical for all the cyclooctynes except the C_2_-symmetric versions, is often a complicating factor. From the conversion plots thus obtained, the second-order rate plots can be calculated according to Eq. (1):

1 $$ kt\, = \,\frac{1}{{[B]_{0} \, - \,[A]_{0} }}\, \times \,\ln \frac{{[A]_{0} ([B]_{0} \, - \,[P])}}{{([A]_{0} \, - \,[P])[B]_{0} }} $$

with *k* = second-order rate constant (M^−1^ s^−1^), *t* = reaction time (s), [*A*]_0_ = the initial concentration of substrate A (mmol/ml), [*B*]_0_ = the initial concentration of substrate B (mmol/ml) and [*P*] = the concentration of triazole product (mmol/ml). Either cyclooctyne or azide can be applied in excess, preferably between 1.2 and 2 (but not stoichiometric). By plotting *t* versus *kt*, calculation of the slope of the resulting (straight) line, gives the reaction rate constant *k*. Better plots are obtained by only including data points up to approximately 50 % conversion. Given the relatively high required concentration of components for fast NMR measurements (typically 10 mM or higher) and the rather time-consuming process before the first measurement can take place (mixing of reagents, insertion in magnet, shimming, scanning), it is clear that NMR has its limitations for very fast SPAAC (>0.5 M^−1^ s^−1^), because more than 50 % of starting material may already be converted at the first measurement and few data points can be taken.

Two alternative techniques for real-time monitoring of (fast) SPAAC processes are UV and IR spectroscopy. For example, rate measurements of SPAAC can be conducted by reaction of a 10–100-fold excess of azide to a low concentration of acetylene (down to 1 × 10^−4^ M) in MeOH. Reaction in this case can be monitored by following the decay of the characteristic absorbance of the acetylene bond, as for example for dibenzoannulated cyclooctyne DIBO [43]. This method may give more accurate rate constants compared to the use of NMR, especially for fast reactions. Importantly, with this large excess of azide over cyclooctyne, the UV spectroscopic method is performed under pseudo-first-order conditions over a wide range of reagent concentrations, making the analysis of second-order kinetic curves more reliable. In case the UV absorption of the acetylene bond is less distinct, as for the aliphatic cyclooctynes, and no other specific UV chromophores can be identified in starting materials or reagents, an IR-based method may be a viable alternative. We recently reported [20] that the substrate-to-product conversion can be directly monitored by integration of the distinct stretch vibration of azide (~2100 cm^−1^). Conveniently, deuterated solvents are not necessary and it was found that IR monitoring could even be executed in 10 % aqueous THF.

**Fig. 8:**
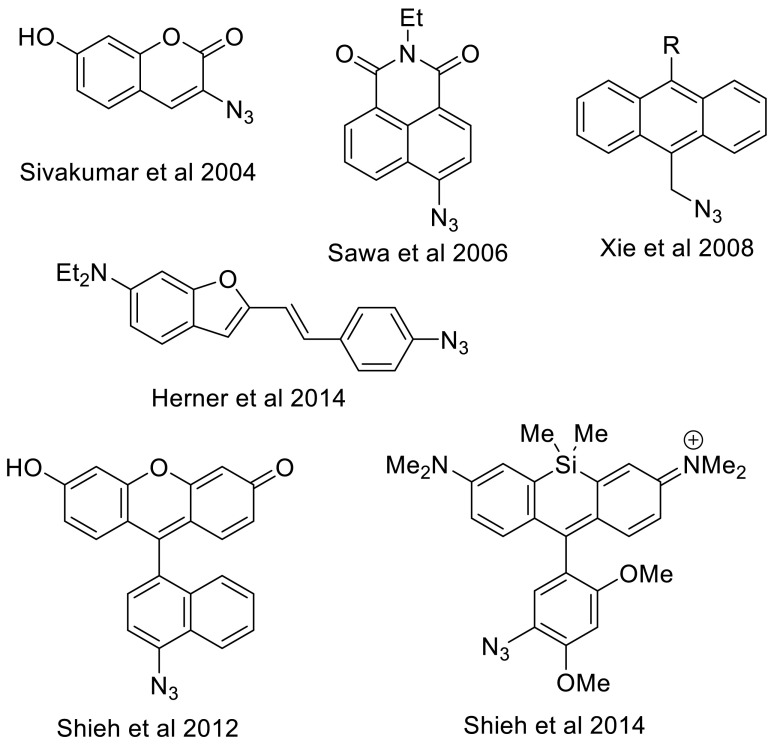
Fluorogenic azide probes for cycloaddition with alkynes

The most sensitive method for determination of reaction rate constants of cyclooctynes is by means of a reaction with a fluorogenic azide substrate [35]. By definition, a fluorogenic SPAAC process involves a reaction between a non-fluorescent alkyne and azide, allowing the ligation of two biomolecules to afford a highly fluorescent triazole product. Besides that, compounds that become fluorescent upon reaction with a chemical reporter and without the need of copper have many attractive features as bioorthogonal probes, such as eliminating the need for probe washout, reducing background labeling, and offering opportunities for monitoring biological processes in real time. The first fluorogenic click reaction based on readily synthesized azidocoumarin derivatives was reported by Sivakumar et al. [53]. Moreover, the photophysical fluorescent properties of coumarines can be strongly enhanced by substitution with an electron withdrawing group at the 3-position and/or an electron donating group at the 7-position. Other early variants of fluorogenic azides involve azidomethylated 1,8-naphthalimide dyes [48] and fluorogenic dyes based on a photoinduced electron transfer (PET) process with anthracene as a fluorophore [63]. It must be noted that the vast majority of fluorogenic probes feature an aromatic azide, which obviously will have a major impact on determination of reaction rate constant in absolute terms, as was for example found by us for a range of BCN derivatives [36]. Other more recent fluorogenic azides, as developed by Shieh et al. [51] and Herner et al. [26] and even a fluorogenic azide substrate for generation of a near-infrared (NIR) triazole dye [52], are depicted in Fig. 8.

**Fig. 9:**
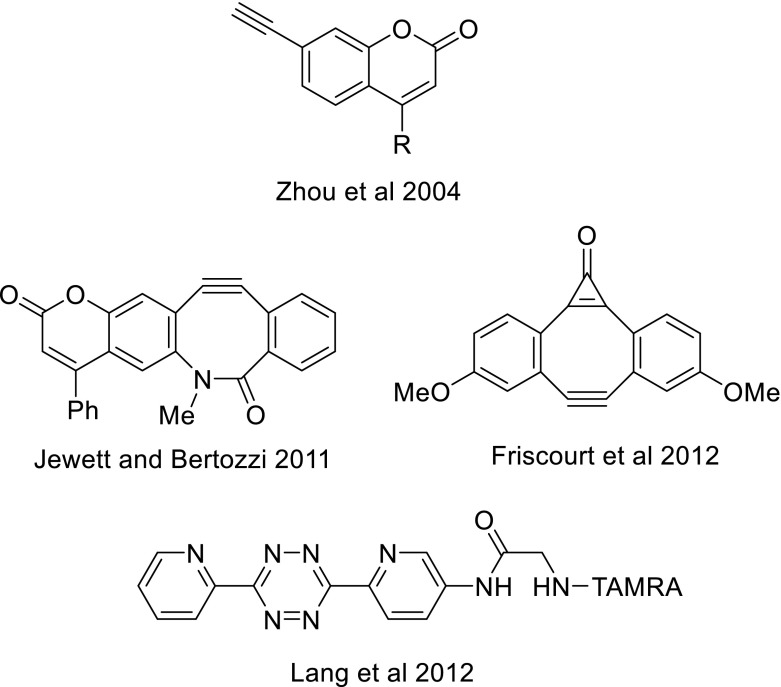
Fluorogenic alkynes for cycloaddition with azides

Notwithstanding the elegancy and versatility of the azide-based fluorogenic probes, in particular to enable the visualization of biomolecules in living systems with biooorthogonal chemistry, it is also clear that for fluorescence detection of an azide label in a biomolecular environment, a complementary fluorogenic alkyne structure is desired. The earliest example of the latter (Fig. 9) was provided in the form of acetylenic derivatives of coumarin by Zhou and Fahrni [65], highly analogous to the azido-coumarin derivatives described by Sivakumar et al. [53]. However, clearly the reaction of a terminal alkyne with an azide requires the undesirable presence of copper(I) to induce the formation of triazole. Two fluorogenic cyclooctyne versions have been developed in the past few years. The first probe was based on annulation of coumarin to BARAC and was developed by Jewett and Bertozzi [29]. Secondly, Friscourt et al. [23] reported that the cyclopropenone derivative of Sondheimer diyne unexpectedly forms a highly fluorescent triazole upon a copper-free reaction with azide. Most recently, Lang et al. [34] showed that a TAMRA-functionalized tetrazine derivative leads to fivefold to tenfold increase in fluorescence signal, similar to earlier reported for reaction with *trans*-cyclooctene and norbornene, upon reaction with BCN.

## Concluding Remarks and Future Prospects

Strain-promoted azide–alkyne cycloaddition (SPAAC), since its inception in 2004, has firmly established itself as a powerful click chemistry tool. The commercial access of starting materials, its ease of operation, the nowadays practical reaction rates, even under high dilution conditions, and the stability of the resulting triazole product, have catapulted SPAAC at the forefront in many research areas in academia and industry. Originally developed for application in bioorthogonal chemistry, SPAAC has proven its value more and more in additional areas of science such as bioconjugation processes, hybrid and block polymers, high-performance and self-regenerative materials, metabolic engineering of biological systems and beyond. One key to the success of SPAAC is the azide component in the reaction, which is easily accessible, small and stable [17]. Nevertheless, recent years have elegantly demonstrated the power of cyclooctyne chemistry beyond cycloaddition with organic azides. Although much of these reactions were known for more than 50 years, mostly by work of Huisgen in the field of 1,3-dipolar cycloadditions, the past decade has witnessed a strong revival of cyclooctyne for development of fast and selective reaction with a range of other alkynophiles. For example, we were to apply [41] strain-promoted alkyne–nitrone cycloaddition (SPANC), a reaction also reported by McKay et al. in the same year [37], for the N-terminal labeling of proteins. Similarly, both our laboratory [27] and Sanders et al. [47] reported the reaction of cyclooctynes with nitrile oxides (SPANOC) leading to isoxazoles, which is a factor ~10 faster than SPAAC and SPANC. Finally, strain-promoted cycloaddition with diazo-compounds is also known [47] as well as reaction of BCN with sydnone [42, 61]. Interestingly, in the field of (4 + 2) cycloadditions, cyclooctynes show reaction rate constants more than a factor 1000 higher than for 1,3-dipolar cycloadditions. It has been known for more than 30 years that aliphatic cyclooctynes undergo extremely fast (4 + 2) cycloadditions with electron-poor tetrazines [3]. The latter process can be tailored to specific reaction rates with electron-rich cyclooctynes like BCN in organic solvents [15] and was found to proceed at a surprisingly high reaction rates >1000 M^−1^ s^−1^ under aqueous conditions when applied for protein labeling [9, 34]. We most recently showed that (4 + 2) cycloaddition of BCN with 1,2-quinone (SPOCQ) proceeds with reaction rates intermediate of azide and tetrazine (~500 M^−1^ s^−1^) and can be applied for rapid labeling of proteins with genetically encoded BCN [10] or formation of gel networks in the order of seconds [30].

Finally, it seems fair to state that SPAAC has now matured into more than just a spectacular click reaction or exciting research tool. As noted earlier, only in the past 5 years more than 100 patent applications have been filed on the use of SPAAC with DIBAC or BCN, with applications in e.g., nanoparticle functionalization, polymer functionalization, genetic encoding, (biodegradable) hydrogels, controlled drug release, oligonucleotide tagging, DNA libraries, peptide arrays, long-acting biopharmaceuticals, radioisotope labeling, and nuclear imaging. Among these, arguably the most prominent application of SPAAC is in the field of the selective and site-specific conjugation of proteins, such as PEGylation, radioisotope labeling, and controlled release of biopharmaceuticals. For example, in the field of targeted anti-cancer therapy alone, a range of pharmaceutical companies (MedImmune, Novartis, Agensys, Sutro, Innate Pharma, Synaffix) are building (part of) their technology around the controlled attachment of highly potent toxins (payloads) to monoclonal antibodies—to prepare antibody–drug conjugates or ADCs—by means of installation of azide into the protein (genetic encoding or enzymatically) followed by highly specific SPAAC. A remarkable position for the process of spontaneous cycloaddition of cyclooctyne and azide, discovered more than 60 years ago out of purely chemical curiosity.
